# Study on Press Formability and Properties of UV-Curable Polyurethane Acrylate Coatings with Different Reactive Diluents

**DOI:** 10.3390/polym15040880

**Published:** 2023-02-10

**Authors:** Woo-Chan Choi, Vishal Gavande, Dong-Yun Kim, Won-Ki Lee

**Affiliations:** 1 Central R&D Center, Dongkuk Steel Mill, Nam-gu, Busan 48481, Republic of Korea; 2 Division of Polymer Engineering, Pukyong National University, Busan 48513, Republic of Korea

**Keywords:** UV-curable coatings, reactive diluents, pre-coated metal, formability

## Abstract

UV-curable coatings have numerous advantages, including environmental sustainability due to 100% solid content, economic feasibility attributable to relatively fast curing time, decent appearance, mechanical properties, chemical resistance, and abrasion resistance. However, UV-curable polyurethane acrylate coatings on metals apparently restrict their engineering applications owing to low mechanical properties and poor thermal stability, giving UV-curable coatings less flexibility and formability. In this study, we evaluated the property change of films according to the type of reactive diluents that lowers the viscosity of UV-curing coatings for pre-coated metal and has a substantial effect on the curing rate, viscoelastic properties, adhesive properties, and flexibility of the film. Moreover, there are many changes in the properties of coatings according to varied curing conditions in order to evaluate the oxygen inhibition phenomenon during the curing process in the atmosphere. In particular, to evaluate the effect of reactive diluents on forming formability, which is the most crucial property for the pre-coated metal, this study used conventional formability tests, such as t-bending or the Erichsen test. Moreover, a cross-die cup drawing mold with a similar form as failure and Safety Zone was utilized in order to obtain clearer information on its actual formability. The analysis on the effect of failure and safety zone on the material used in press forming was conducted by assessing limit punch height and forming a limit diagram of the manufactured film according to varied reactive diluents.

## 1. Introduction

Pre-Coated Metal (PCM) coatings are applied to a uniform panel prior to pre-coating and metal processing, making it substantially more productive than coating complicated shapes of metals. The coated steel panels are milled into various products by cutting, forming, and assembling. It is a product fabricated in the way paints with corrosion resistance and formability are coated on electro-galvanized steel or galvanized steel sheet with 0.3~2.0 mm of thickness. PCM coatings are primarily applied to improve the appearance of refrigerators, washing machines, microwave ovens, and air conditioners. It has broad applications as it does not need additional post-processing, such as coating after processing [1,2,3,4,5].

Most of the coatings employed for PCM contain organic solvents. Solvent-typed coatings typically have the benefits of affordable prices and superb property. However, ample volatile organic solvents generated during the manufacturing process cause environmental problems [6]. These are emerging as crucial issues across industries applying the coating process as well as the PCM manufacturing process. In order to deal with such social issues and environmental regulations, many corporations and institutions are actively researching the development of eco-friendly coatings [6,7,8,9,10]. The eco-friendly coatings currently being developed with the goal of minimizing the amount of organic solvents include water-borne types, ultrahigh solids (UHS), powder coatings, etc. However, the properties of eco-friendly coatings are somewhat inferior to those of organic solvent-based coatings, so the latter are more widely used in a range of industries. In order to solve such problems, it is absolutely essential to develop coatings ensuring eco-friendliness, good mechanical and adhesive properties, improved operating efficiency, and productivity.

**Figure 1:**
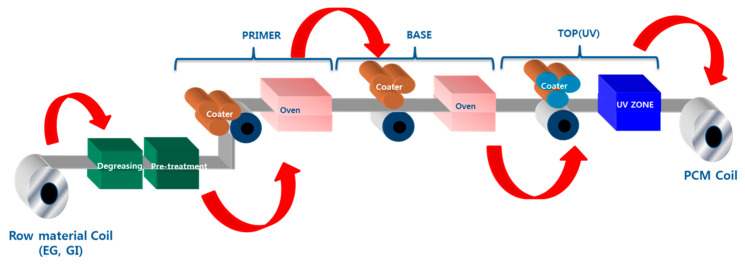
Schematic process of UV curing coatings for pre-coated metals.

UV-curable coatings, which are recently emerging as a new PCM coating system, are eco-friendly because it does not contain solvent [4]. Additionally, UV-curable coatings show higher productivity and require less energy compared to the existing process, a thermal-curable one, due to the rapid curing mechanism. Figure 1 shows the manufacturing process of UV-PCM. To apply UV-curable coatings to the PCM process, however, there lies an obstacle to be solved [11]. Poor mechanical and thermal properties, poor formability, and flexibility limit their application in PCM industries, where flexibility matters, so there remains a difficulty in actual applications. Moreover, the rapid curing velocity causes wrinkles and poor bonding owing to the gap in shrinkage rate between UV-curable coatings and materials [12,13]. Meanwhile, UV-curable coating does not contain solvents but has a high viscosity, thus requiring a good deal of reactive diluents to improve productivity. Therefore, it is hard to embody all features of coatings required in PCM. Using proper reactive thinners is crucial in which reactive diluents can affect cured composite, including improving flexibility and adhesive property, strength, curing velocity, operating conditions, and viscosity.

Acrylate oligomers are linear molecules containing two double bonds. Their viscosity is higher and causes difficulties in use. Therefore, reactive diluents must be used. Seo et al. reported on the improved mechanical properties of UV-curable coatings by using hyperbranched polyurethane acrylate with two different reactive diluents, methyl methacrylate (MMA) and trimethylolpropane triacrylate (TMPTA) [14]. Cheon et al. reported the properties of the UV-curable polyurethane acrylate coatings by varying the kind (IBOA and trimethylolpropane ethoxylate triacrylate (TMPEOTA)) and content of the reactive diluents [1]. They reported that an increase in TMPEOTA content results in an increase in the T_g_, storage modulus, and mechanical properties. Antibacterial UV-curable coatings based on the polyurethane acrylate using eugenol as a reactive diluent were studied by Bednarczyk et al., who studied the influence of the amount of eugenol on the chemical, physical, thermal, and mechanical properties [15]. Oh et al. reported that cardanol-based acrylates as reactive diluents for UV-curable coatings. They reported that the bio-derived cardanol-based acrylates in UV-curable films exhibit a low dilution effect, low reactivity, low T_g_, and high thermal stability compared to petroleum-based diluent phenoxyethyl acrylate (PHEA) [6].

In this study, our team has evaluated the changes in coating properties by introducing many types of reactive diluents, which lower the viscosity of UV-curing coating along with the effect of these diluents on the curing velocity, mechanical properties, adhesive properties, and elongation properties of coating films. Additionally, the properties of coatings according to varied curing conditions were compared and studied to confirm whether oxygen existed or not in the atmosphere interrupts the curing process in the UV curing reaction. In particular, to evaluate the effect of reactive diluents on formability, one of the most crucial properties for PCM, this study used conventional flexibility tests such as T-bending or the Erichsen test. These tests enable us to predict faulty processes but do not present clear standards for cracks or necking caused by forming defects in press forming. Moreover, these tests make it difficult to evaluate the complex formability that can occur during press forming. A Cross-Die Cup drawing mold with a similar form as the failure and safety zone was utilized in order to obtain obvious information on its actual formability. The analysis on the effect of failure and safety zone on the material used in press forming was conducted by assessing the limit punch height (LPH) and forming limit diagram (FLD) of the manufactured film according to varied reactive diluents. The drawing strain of the film was also analyzed via automated strain analysis and measurement environment (ASAME).

## 2. Experimental

### 2.1. Materials

Two oligomers, 100% solids aliphatic urethane diacrylate (UA9359) and a 6-functional aliphatic urethane hexacrylate (Ebecryl 1290), were received from SK CYTEC and their physical characteristics were shown in Table 1. Isobornyl acrylate (IBOA, Nippon Shokubai, Osaka, Japan), isobornyl methacrylate (IBOMA, Nippon Shokubai), tripropylene glycol diacrylate (TPGDA, Miwon, Anyang, Republic of Korea) and 1,6-hexanediol diacrylate (HDDA, Miwon) were used as reactive diluents. The photoinitiator was 1-hydroxy cyclohexyl phenyl ketone (Irgacure 184, Ciba). A modified polyether polysiloxane (EFKA-3035, EFKA), leveling agent, was used to provide wetting and smoothness of the coating. All materials were used as received.

### 2.2. Method

The formulations of the coatings used in this experiment are shown in Table 2. Different reactive diluents (Figure S1 (ESI†)) were added to the formulation. After mixing the ingredients (Oligomer, reactive diluent, photoinitiator, and leveling agent) of each formulation, the mixture was stirred at 2000 rpm for 20 min in a dispersion disk having a diameter of 30 mm using a high-speed disperser (Dispermat CV, VMA) at room temperature. After dispersion, stabilization was carried out for 1 h. UV curing steel sheet specimens were prepared by using bar coating with primer coating (5 μm, Bar-coater No. 18) and top coating (15 μm, Bar-coater No. 32) with a polyester melamine coating on galvanized steel (0.6 mm thickness). The curing conditions were determined by setting the peak metal temperature (PMT) to 210 °C using an automatic exhaust oven (TSAS-103A, Taesung Engineering, Anyung, Republic of Korea). Coatings with different reactive diluents were coated on the prepared steel plate specimens to a thickness of 30 μm using bar coating (bar-coater No. 30) and then cured in a UV chamber equipped with a high-pressure mercury lamp (160 w/cm, 2 × 2 lamp, SMV-5000, Sei Myung Vactron, Gyeonggi-do, Republic of Korea), the amount of UV light was 2000 mJ/cm^2^, and the amount of the light was measured using a light amount measuring device (UV Map, EIT 2.0, Sterling, VA, USA). The viscosity of the UV-cured coating was measured using a # 4 spindle of programmable viscometer (DV-II + viscometer, Brookfield) at 25 °C and the results are shown in Table S1 (ESI†). All of the reactive diluents showed excellent dilution power, and HDDA was shown the lowest viscosity. 

### 2.3. Characterizations

#### 2.3.1. FT-IR

FTIR spectrum was employed to identify the curing characteristics of the coatings. The peaks were measured before and after UV irradiations using a Fourier transform infrared spectrometer (FTIR, FTS-165, BIO RAD, Hercules, CA, USA) after thinly coating the coating solution on the KBr plate. Sixty-four scans were averaged to each sample, and the wave number range was set from 4000–500 cm^−1^.

#### 2.3.2. Rigid-Body Pendulum Testing (RPT)

The curing behavior of the UV-curable coatings was characterized using a rigid-body pendulum physical property testing instrument (RPT-3000, A&D Co., Tokyo, Japan) and UV-irradiation Instrument (EX250, HOYA-SCHOTT). The curing behavior was characterized by identifying the movement of a knife-type pendulum on the coated steel substrate as a function of UV irradiation. The oscillation pattern of the pendulum is associated with change in surface properties, such as chemical or physical networking. To examine curing characteristics, the UV curable coatings were coated on the steel substrate (50 mm × 20 mm × 0.3 mm) using a 30 μm coating tool (PCT-030, wet gauge: 30 μm).

#### 2.3.3. Dynamic Mechanical Analysis (DMA)

Dynamic viscoelasticity characteristics were measured employing a dynamic mechanical analyzer Q-800 (TA Instruments, Inc., New Castle, DE, USA). The test conditions were tensile mode, the frequency was set to 1 Hz, the strain was set to 0.1%, and the temperature was measured at 3 °C/min in a temperature range of −50 to 150 °C. The thickness of the specimens was maintained at 0.1 mm, 8 mm width, and 35 mm length.

#### 2.3.4. Cross Cutter Erichsen Test (CCET)

To identify the adhesion properties of the UV-cured coatings (with various diluents) on the PCM sheets, the cross-cut Erichsen test was conducted by the adhesion tester with cross-cut 100 squares with 1 mm of width and 1 height by a Crosshatch cutter. After that, the squares with 6 mm of width and height were processed by the Erichsen Testing machine. Since the 100 cells were cross-cut with a gap of 1 mm and cross-cut tape test was carried out with a strong adhesive tape 4 times in each direction [16]. After measuring the experiment value five times, the mean value was calculated (ASTM D-3359).

#### 2.3.5. Gloss

Gloss test is used to evaluate the level of mirror direction reflection of film, and the gloss was assessed by a micro-tri-glossometer (ASTM D-523).

#### 2.3.6. Pencil Hardness

Hardness is one of the crucial mechanical features necessary for the coated layer to play the role of protecting the product against external pressure. The hardness was determined by determining scratch resistance with a CT-PC2 (CORETECH, Gyeonggi, Republic of Korea) pencil hardness tester, as per the standard ASTM: D-3363 employing the softest to hardest pencil (6B–9H) with a 1000 g loading and an angle of 45°. The test was carried out at 23 ± 2 °C on the horizontal surface of the film as the pencil was moved over the coated substrate.

#### 2.3.7. T-Bending Test

T-bending test evaluates the formability and adhesive property of films by artificially bending the PCM used for construction materials and home appliances. The formability test was carried out via a 1/4 in. vise bending tester (ASTM D4145).

#### 2.3.8. Erichsen Test

To determine the adhesion and drawing of coatings after deformation, the Erichsen test was conducted by inserting a punch with the shape of a 20 mm diameter sphere into the cured coating. The larger the compression size set to Erichsen is, the larger the drawing of the film (ASTM E-643-09).

#### 2.3.9. Impact Resistance

The impact resistance test is to evaluate the strain of base material and film by putting a sudden impact on to film. It is conducted by dropping the ball under the condition of a maximum of 500 mm of drop height, 1 kg of dropped load, and ball diameter of 0.5 inches to identify the extent of rupture/peeling of the film (ASTM D 2794).

#### 2.3.10. Chemical Resistance

The alkali and acid resistance of the coatings were evaluated by immersing half of the film in the solution of 5.0% of NaOH and CH_3_COOH at a temperature of 50 °C for 24 h, and the extent of swelling or discoloration was checked with the aided eye (JIS K 5400).

#### 2.3.11. UV Light Resistance

This test was carried out by irradiation of 20 W × 20 cm × 24 h on the film or paint via UV-B ramp manufactured by Toshiba, ΔE, was assessed (JIS K 5400).

#### 2.3.12. Formability

**Figure 2:**
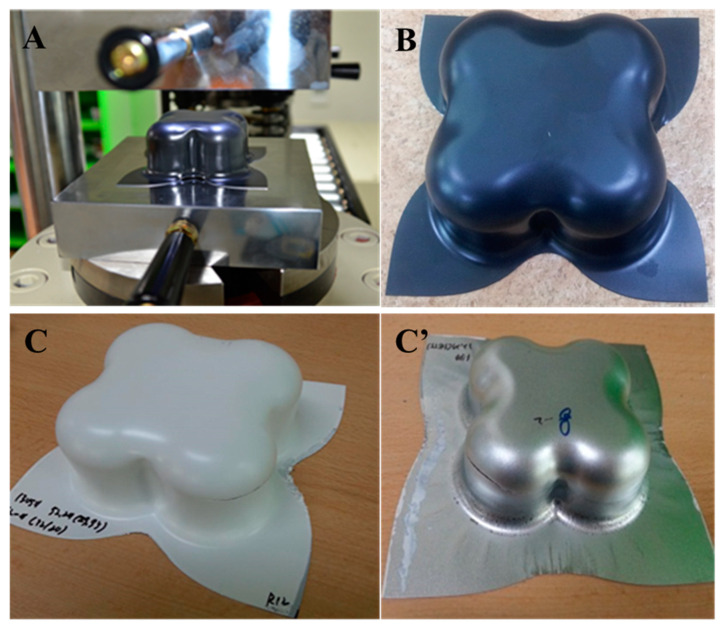
Cross-die cup drawing mold and press for PCM: (**A**) Cross-die drawing mold, (**B**) cross-die drawing sample (mom-fractured), (**C**,**C’**) cross-die drawing sample (fractured).

**Figure 3:**
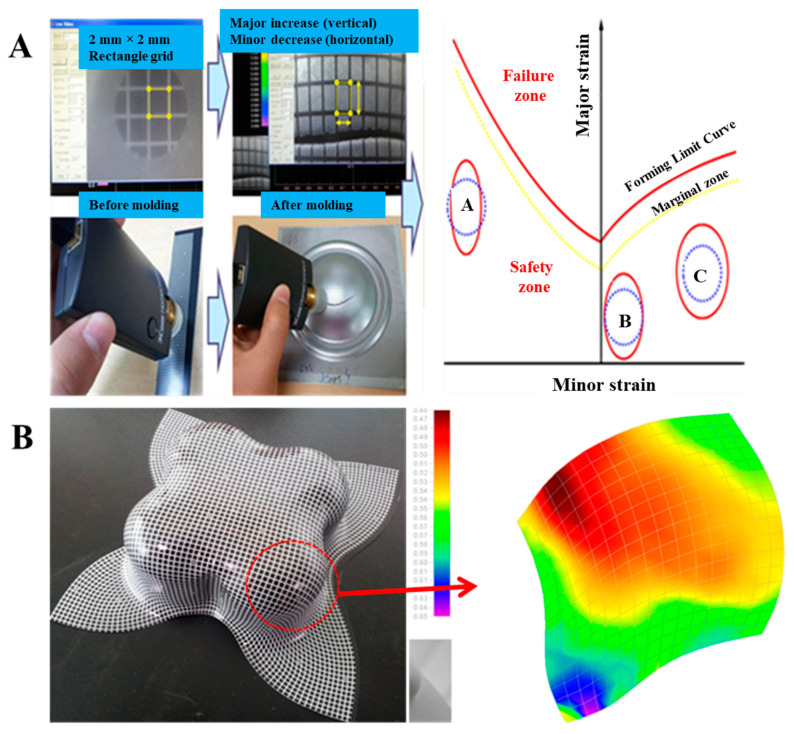
Failure and safety zone in FLD and ASAME strain analysis: (**A**) Failure and safety zone in FLD and (**B**) cross-die test sample ASAME target model strain analysis.

The effect of failure and safety zone on the materials used in press forming was analyzed by the Limit Punch Height (LPH) and Forming Limit Diagram (FLD) of the fabricated films according to the varied reactive diluents. The drawing strain of the film was also analyzed via ASAME, while LPH, which is intended to measure the maximum height at which forming material is possible before it is fractured, represents quantitative features according to the compression and tension. As shown in Figure 2, measurements were made using a cross-die drawing mold. The cross-die drawing sample was produced using a deep drawing machine (USTM, 150T-WF500). The experimental conditions were 10 t of blank holding force and 20 mm/min punch speed. As shown in Figure 3A, FLD is shown using a cross-die drawing mold. FLD is an important tool for determining whether a material can be formed or not. It provides criteria for failure and Safety Zone in press forming. FLD analysis can predict the forming limit of PCM. The vertical axis represents the major strain, and the horizontal axis represents the minor strain. A is the uniaxial tensile-compression deformation mode region, B is the plane-deformation mode region, and C is the biaxial tensile-deformation mode region. The formability of a product can be evaluated by plotting on FLD the strain of each part of the test specimen formed by die with lattice marked on it. ASAME, employed to find out with ease of cracks arising during the process, enables us to measure easily the strain distribution of formed material in press forming on cross-section or three-dimension regardless of place. It is possible to identify the strain distribution by using a range of colors via three-dimensional shape information. Purple indicates a low reduction rate of the coating thickness, and red suggests a high reduction rate [17,18,19].

#### 2.3.13. Gel Content

Gel contents of the UV-cured films were calculated using Soxhlet extraction. The known weight of the coating film is immersed in toluene for 24 h and then dried at 60 °C for 3 h. Gel content was determined from the weight ratio of the specimen after and before Soxhlet extraction.

## 3. Results and Discussion

**Figure 4:**
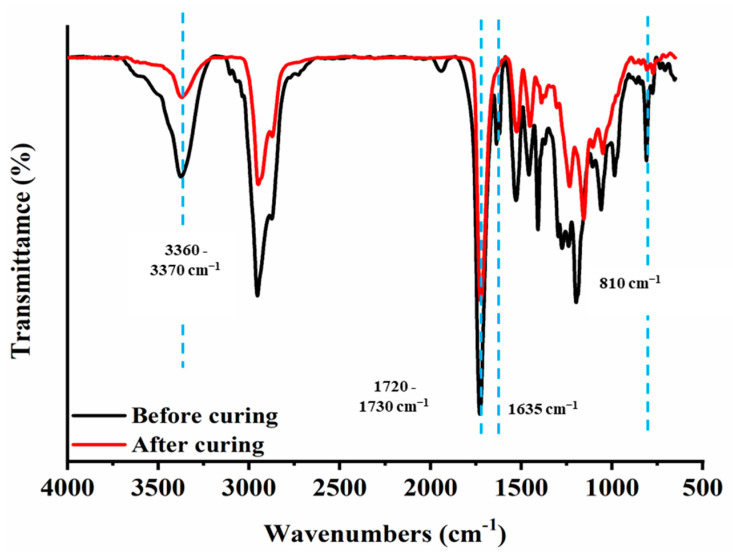
FT-IR spectra of P-IBOA coatings (before and after curing).

FTIR analysis was conducted to evaluate a change in IR attribute peak before and after curing. Figure 4 depicted the spectra of P-IBOA coating formulation before and after UV-curing. Before the curing of P-IBOA formulation, there was a strong absorbance of acrylate group C=C at 810 cm^−1^ and 1635 cm^−1^. After curing, the acrylate group (C=C) peaks at 1635 and 810 cm^−1^ disappeared in the spectrum cured PUA film owing to the crosslinking reaction accompanied by photopolymerization. Moreover, the attribute peaks at 3360–3370 cm^−1^ (N-H stretching) and 1720–1730 cm^−1^ (carbonyl stretching) were observed after photopolymerization. It indicates that the urethane acrylate oligomer was polymerized successfully into polyurethane acrylate films. Similarly, after curing, the acrylate group (C=C) peaks at 1630 and 810 cm^−1^ disappeared in the spectrum cured P-IBOMA, P-HDDA, and P-TPGDA films owing to the crosslinking reaction accompanied by photopolymerization. There are no significant changes in the characteristic peaks.

**Figure 5:**
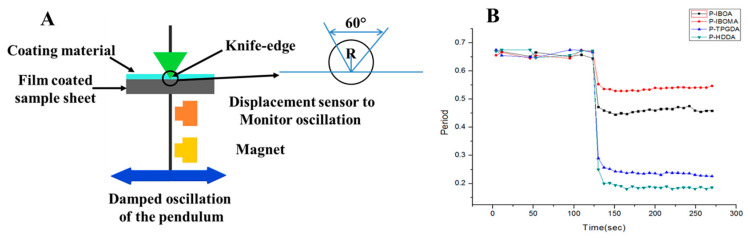
(**A**) Rigid body type pendulum in RPT instrument: Knife edge-type for the curing process. (**B**) Curing behavior of UV-curable coatings.

Curing behavior is a significant characteristic for the PCM, and it provides crucial information on curing conditions. Rigid-body pendulum physical property testing instrument (RPT) was used to identify the changes of property arising from curing from liquid to solid, which induces damped oscillation [20]. The RPT detects the curing behavior of the coatings on the steel substrate as a function of UV irradiation. The curing density of coatings can be estimated indirectly by assessing vibration at the point where curing proceeded to the free-damped oscillation of the pendulum. The bigger the slope is, the faster the reaction velocity is facilitated, and the deeper the depth, the higher the crosslink density. At the time of UV irradiation, the lower the period value is, the higher the crosslink density. Figure 5 represents the UV-curing behaviors of coatings measured from RPT. Mono-functionality methacrylate monomer, IBOMA, showed lower crosslink density than IBOA, which is attributed to the oxygen existing in the atmosphere. Another reason is that methacrylate monomers have a slower curing velocity than acrylate monomers due to the steric hindrance of CH_3_. However, multifunctional reactive diluents P-TPGDA and P-HDDA showed higher crosslinking density than IBOA and IBOMA. It was accepted that higher functionality of reactive diluents increases rapid curing rate and a high degree of crosslinking [21].

**Figure 6:**
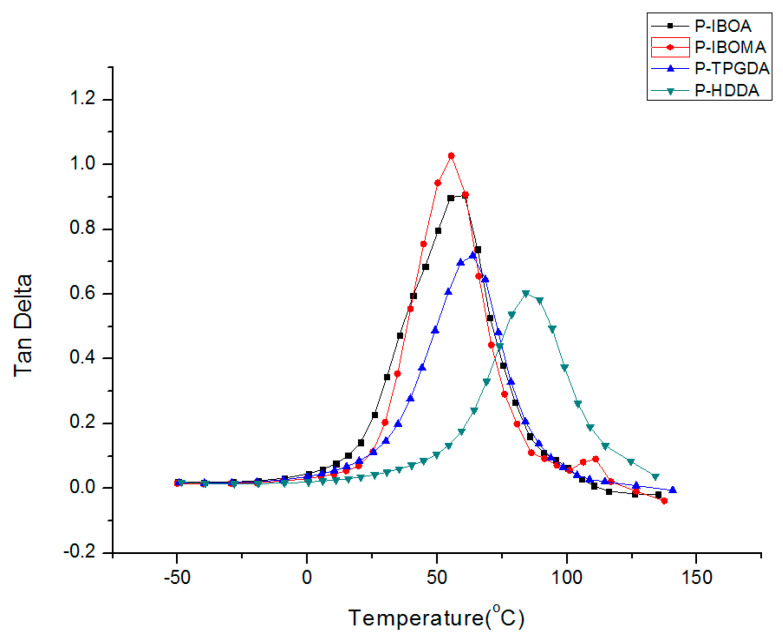
DMA properties of UV-cured PUA coating films.

Dynamic mechanical analysis (DMA) is a suitable and effective technique to study the viscoelastic properties of UV-cured polymeric materials. The DMA data concede observations of changes in loss and storage modulus, glass transition temperature (T_g_), and cross-link density of paints and coatings. Figure 6 represents the tan δ of the UV-cured films as a function of temperature. The tan δ values of mono-functionality reactive diluents, P-IBOA and P-IBOMA, have relatively higher than those of P-TPGDA and P-HDDA, which are multi-functionality reactive diluents. The T_g_ of P-IBOA and P-IBOMA is less than both P-TPGDA and P-HDDA, which provides low stiffness and high softness to the polymer coatings. The T_g_ of UV-cured coatings was increased as the functional groups increased. Mono-functional reactive diluents lead to a decrease in T_g_, modulus, and ductility, while multifunctional reactive diluents lead to an increase in T_g_, modulus, and ductility because a high degree of functionality of reactive diluents leads to a high reaction rate and a high degree of crosslinking density [22]. P-TPGDA and P-HDDA-based coatings have extra reactive sites in the backbone for crosslinking as compared to monofunctional reactive diluent-based coatings. The multifunctionality also can lead to a low final degree of conversion because early gelation of the UV-irradiated sample restricts the mobility of the reactive sites and can lead to the glassy nature of the polymer coatings, resulting in the increase in T_g_ of the UV-cured coatings [23].

**Figure 7:**
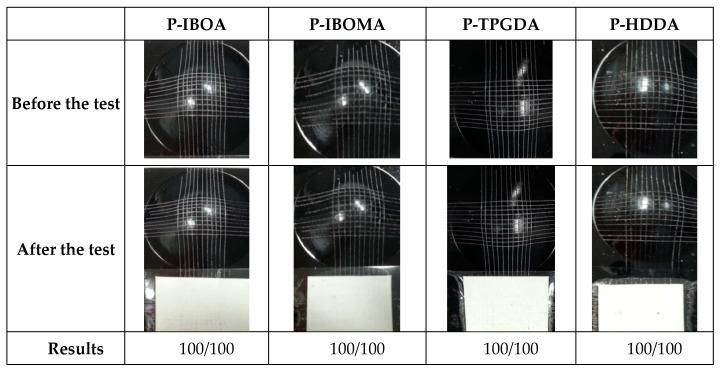
Cross-cutter Erichsen test (C.C.E.T) of UV-cured PUA coating films.

To determine the adhesion and formability of the coatings after deformation, the cross-cutter Erichsen test was carried out by a Cross-cutter Erichsen tester. All of the UV-cured coating films show good adhesion and good formability to PCM. Due to the introduction of a coating agent, modified polyether polysiloxane in the formulation, the wetting properties of the film were increased as the reactive diluents used in the formulation were eroded on the surface of the coating film, thus increasing the toughness of the surface of the adherend layer. However, in this study, the Erichsen test was conducted until the deformation of 6 mm. As shown in Figure 7, all UV-cured coating samples had shown deformation excluding any sign of cracks.

**Figure 8:**
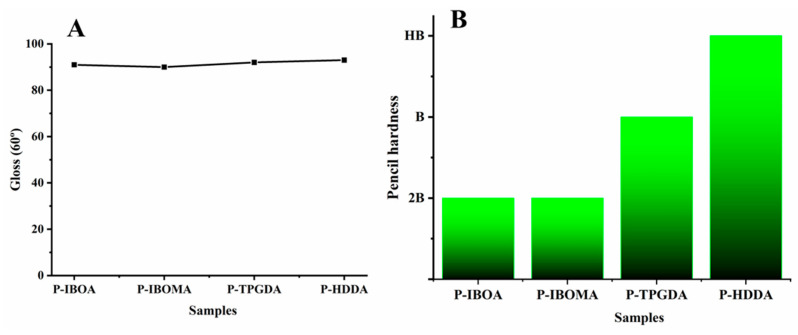
(**A**) Gloss values at an angle of 60° and (**B**) pencil hardness of UC-cured PUA coating films.

Gloss is one of the substantial properties for coating applications, as determined by the micro-tri-glossometer. Figure 8A shows the effect of the reactive diluents on the gloss values of the surface of the cured bonds, whereas the hardness of P-IBOMA turned out to be a low grade of 2B, so it is hard to be applied to the actual PCM applications because of the very low-hardness films. All of the UV-cured PUA coatings with different reactive diluents depicted similar gloss values at the angle of 60°. As shown in Figure 8B, P-HDDA showed the maximum hardness due to the high crosslinking density.

**Figure 9:**
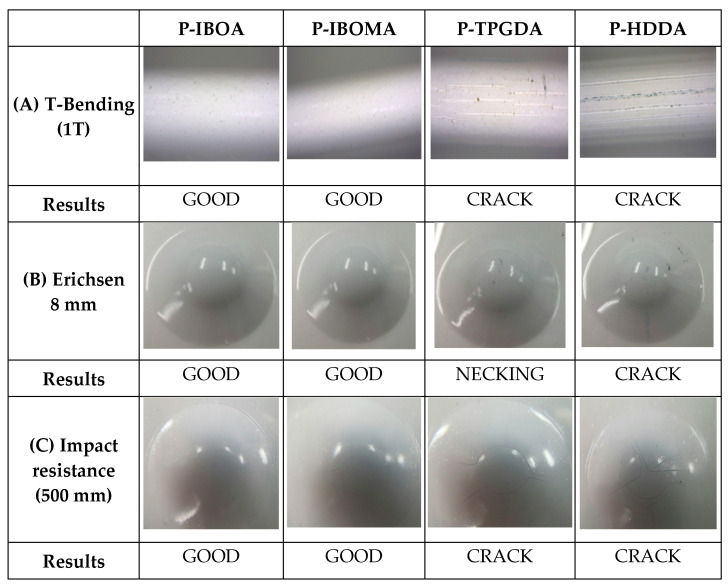
(**A**) 1T T-bending, (**B**) Erichsen (8 mm), and (**C**) impact resistance (500 mm) test images of UV-cured PUA coating films.

The t-bending test has been accustomed to evaluate the formability of distinct coating systems in a cooperative way. The T-bend test results are registered in the form ‘xT’ where the x denotes the level of T-bend which a coating can sustain without failure by through-thickness cracking of the coating or loss of adhesion [24,25]. A 0T T-bend relates to a spacer thickness of 0× the sheet metal thickness, a 1T T-bend relates to a spacer thickness of 1× the thickness of the sheet metal, and so on. The T-bend level in which a coating can tolerate without failing by cracking is compared between coatings, or a minimum T-bend level which a coating must pass for an application is defined. As shown in Figure 9, the 1T-bending test was carried out on all of the UV-cured PUA coating films. As shown in Figure 9, a crack occurred in P-TPGDA and P-HDDA, whereas there was no sign of cracks in P-IBOA and P-IBOMA. However, as per 1T T-bend test, P-IBOA and P-IBOMA were shown quite good results with no cracks, while with 3T T-bend test for P-TPGDMA and 4T T-bend test for P-HDDA coating films was shown no cracks (Figure S2 (ESI†)).

To identify the adhesion of coating after deformation, the Erichsen Test was conducted by Erichsen Cupping Tester. In the PCM industries, the standard index of deformation is 6 mm. It indicates that there should not be any sign of cracks in the coating after 6 mm of deformation. In this study, the Erichsen test was carried out until the deformation of 8 mm. As shown in Figure 9B, necking occurred in P-TPGDA, and crack occurred in P-HDDA, whereas the 8 mm Erichsen drawing of P-IBOA and P-IBOMA turned out to be decent while in the case of P-HDDA sustained deformation after 7 mm with small necking (Figure S3 (ESI†)).

All coatings are subjected to impact damage during their fabrication and lifecycle. This test method for impact resistance has been considered it appropriate in estimating the impact resistance to the coatings. It provides meaningful data for rapidly deforming by the impact on coating films and their substrate for observing the deformation effects. As shown in Figure 9C, P-IBOA turned out to be the most superb, and P-IBOMA did not crack but had a scratch on its surface due to its softness, while P-TPGDA and P-HDDA sustained impact resistance up to 300 mm and 200 mm, respectively (Figure S4 (ESI†)).

**Figure 10:**
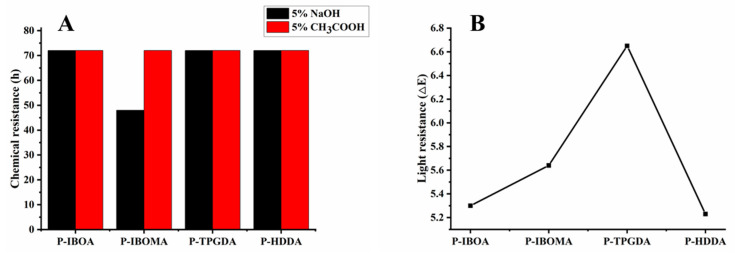
(**A**) Chemical resistance and (**B**) UV-light resistance of UV-cured PUA coating films.

As in Figure 10A, P-IBOMA was a little swollen in the alkali resistance test, and the other films showed no particular sign. These results indicate that a low cross-linking density makes solvent molecules easy to penetrate in film networks, swelling the film in the organic medium. All films showed good results against the acid resistance test. As shown in Figure 10B, P-HDDA turned out to have the most superb performance compared to other UV-cured coating films.

**Figure 11:**
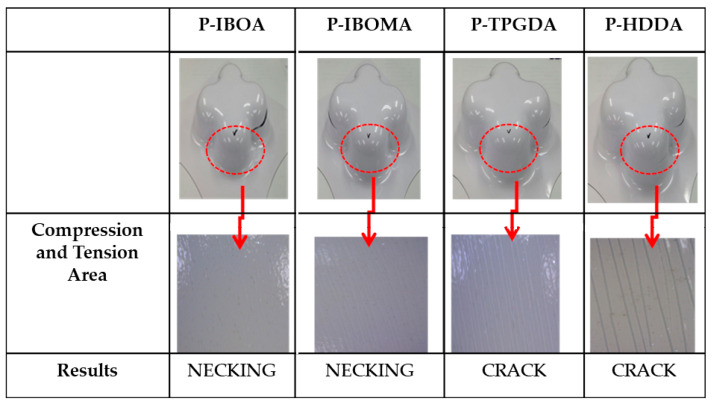
Compression and tension images of UV-cured PUA coating films.

**Figure 12:**
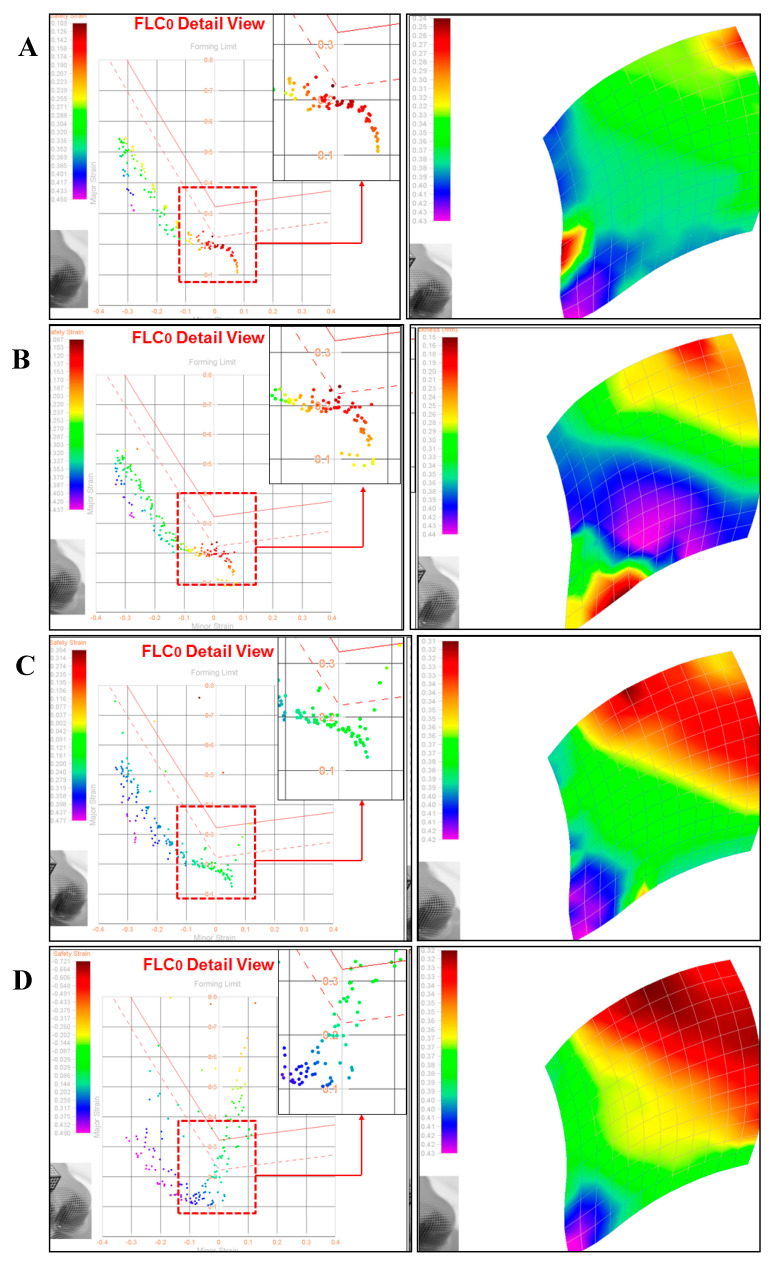
ASAME strain signature for UV-cured PUA coating films: Result of the sample (**A**) P-IBOA, (**B**) P-IBOMA, (**C**) P-TPGDA, and (**D**) Result of sample P-HDDA for draw depth 40 mm.

PCM undergoes forming process for the final product, and the formability of a product is a crucial factor in determining the commercialization of a product. The formability of PCM is basically evaluated via T-bending and Erichsen tests, which do not provide information on the limit of formability according to various compression and tension, so there remains a limit to actual application. As mentioned above, cross-die drawing mold is an effective tool to analyze the formability of PCM. As mentioned above, cross-die drawing mold is an effective tool to analyze the formability of PCM. Table 3 and Figure S5A (ESI†) showed the result of assessing LPH using a Cross-Die Cup drawing mold [26]. Considering the highest results of hardness value and T-bending tests, P-IBOA is likely to have superb press formability, but there is a sign of necking occurring at the drawing part with compression and tension, which was not identified in T-bending or Erichsen tests. Figure 11 and Figure S5B (ESI†) indicated the assessment of the reduction rate of film thickness at the part with the highest compression and tension during the forming process. P-IBOA shows the lowest reduction rate, which is located within the safety zone against forming a limit curve. Referring to the ASAME strain analysis, a large portion of P-HDDA and P-TPGDA assumes red color representing the high strain in strain distribution, as depicted in Figure 12. However, press forming is estimated to work against P-HDDA and P-TPGDA.

### Film Properties by Curing Atmosphere

**Figure 13:**
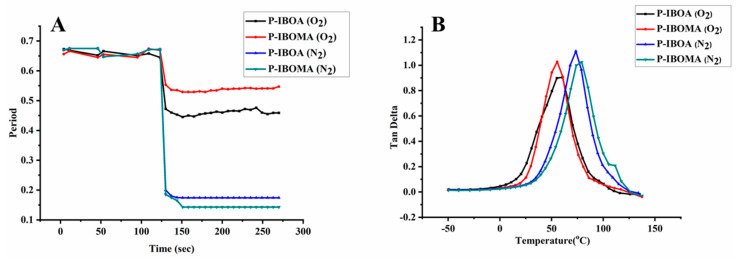
(**A**) Curing behavior and (**B**) Tan δ values of UV-curable PUA coating films in the O_2_ and in N_2_ atmosphere.

The mono-functional P-IBOA and P-IBOMA showed superb results in T-bending or the Erichsen test, the typical processing test, but in press forming properties were absolutely lacking. The much flexibility in coatings and low hardness caused the compression-tension part to have necking or crack, and scratches occurred at some parts of the edge of the film since the film was abandoned. Furthermore, the multi-functional P-HDDA and P-TPGDA had cracks due to a lack of flexibility, which is the most crucial for PCM, so their applicability to PCM is not competent. PUA is used to cure by free radical polymerization. Within the accelerated photopolymerization mechanism upon UV exposure, photoinitiators are triggered to produce radicals. Photopolymerization and crosslinking reactions eventuate suddenly as PUA oligomers reacted with free radicals to develop consecutively larger free radicals. By dominating the samples to different UV conditions, distinct material properties were achieved due to variations in the degree of cure. Since it is a radical polymerization process in UV-curing system, the curing of films is easily interrupted by oxygen in the air [27,28,29,30]. Free radicals originating amid UV irradiation of acrylates are short-lived species which are terminated rapidly after contact with oxygen from the surrounding atmosphere. It is questionable that further reaction witnessed after UV-curing is owing to the same curing mechanism through the reaction of trapped free radicals. The phenomenon of hindering curing response on the film surface by oxygen generates the lowered property of the film surface. This oxygen-inhibition phenomenon can be solved by curing it in nitrogen or CO_2_ atmosphere or by wax coating [27,31,32,33,34]. Therefore, simple verification was conducted by curing the PUA in a nitrogen atmosphere (N_2_) for removing oxygen that hinders the curing process, and the changes in the properties were evaluated. As shown in Figure 13A, the crosslink density in the N_2_ atmosphere was higher than in the O_2_ atmosphere. Moreover, contrary to the case of being cured in the N_2_ atmosphere, P-IBOMA showed higher crosslink density compared to P-IBOA. That is because methacrylate monomer shows a slower curing response compared to acrylate monomer due to oxygen inhibition reactions, and crosslink density is low due to steric hindrance of CH_3_ [35,36,37,38]. For proving this, the gel content was calculated of the UV-cured coating films by immersing them in toluene solvent for 24 h. The weight changes can be calculated by following Equation (1).

% gel = {(Wo − W)/Wo} × 100(1)

where, Wo is the weight of the test specimen before immersing in the toluene solvent, and W indicates the weight of the test specimen after immersing in the toluene solvent. Table S2 (ESI†) represents the gel content was observed 3.5~4.5% less in O_2_ atmosphere curing than in N_2_ atmosphere curing. This result suggests that the test specimens cured in N_2_ atmosphere or low oxygen phenomenon can be lessened in UV-curing, and it is possible to obtain the coatings with improved properties and hardness. As shown in Figure 13B, the DMA data allow observations changes when cured at O_2_ and N_2_ atmosphere. Tan δ and T_g_ value was higher than they were in the atmosphere, suggesting the high toughness of resin, so the coating is estimated to have good flexibility and physical properties.

Additionally, the adhesive properties, pencil hardness, and UV-light resistance of the UV-cured coating films in the N_2_ atmosphere were better than the UV-cured coating films in the O_2_ atmosphere (Figure S6 (ESI†)). Furthermore, Figure S7 (ESI†) represents the T-bending (1T), 8 mm Erichsen test, and 500 mm impact results were quite superior in UV-cured PUA coated films (P-IBOA and P-IBOMA) in N_2_ atmosphere than UV-cured PUA films in O_2_ atmosphere.

Table S3 (ESI†) and Figures S8 and S9 (ESI†) represent the LPH of P-IBOA and P-IBOMA films in the N_2_ atmosphere, and the reduction rate of film thickness and it performs better in the N_2_ atmosphere than in O_2_ atmosphere. Figure S10 (ESI†) shows that, in the ASAME analysis, a large portion becomes green, suggesting high strain in the films that result in good press forming for the N_2_ atmosphere-cured P-IBOA and P-IBOMA films.

## 4. Conclusions

Mechanical properties, adhesive properties, and press formability are the most crucial properties of the pre-coated metal industries. UV-curable PUA coatings were formulated using different types of reactive diluents for the formulation of UV-curable PUA coatings for PCM. In this study, we have evaluated the mechanical, adhesive, and press formability of the UV-cured PUA coatings, which include different types of reactive diluents. When multi-functional reactive diluent was used in UV-curable PUA coatings, mechanical properties and chemical resistance were excellent, but flexibility was vulnerable. In contrast, when monofunctional reactive diluent was used, the flexibility was quite good, but the low crosslinking density of the coating generated a scratchy surface, and in press forming and the deep processing, necking, or crack occurred at the compression–tension area. Afterward, monofunctional diluent-based UV-cured PUA coatings were cured in the nitrogen atmosphere to improve surface hardness. It showed superb mechanical and chemical resistance and press formability compared to the UV-cured PUA coatings in the O_2_ atmosphere.

However, due to their excellent formability, mono-functionality reactive diluents-based PUA coatings should be used to apply UV-curable PUA coatings to PCM; however, the low hardness of the coating films causes a variety of problems in their applications. This study revealed that if oxygen hindrance is mitigated in film curing, it would be possible to keep flexibility and press formability, which are the most crucial properties in PCM.

## Figures and Tables

**Table 1:** Physical characteristics of oligomers used in this study.

Oligomer	Viscosity (mPa·s)	Density (g/cm^3^)	Molecular Weight	Functionality	Solid
UA9359 ^a^	20,000 (25 °C)	1.12	1000	2	100%
Ebecryl 1290 ^b^	2000 (60 °C)	1.19	1000	6	100%

^a^ Aliphatic urethane diacrylate oligomer. ^b^ Aliphatic urethane hexacrylate oligomer.

**Table 2:** Formulations of the UV-curable coatings.

Components		P-IBOA	P-IBOMA	P-TPGDA	P-HDDA
**Oligomer**	**UA9359 ^a^**	55	55	55	55
	**Ebecryl 1290 ^b^**	5	5	5	5
**Diluent**	**IBOA ^c^**	34	-	-	-
	**IBOMA ^d^**	-	34	-	-
	**TPGDA ^e^**	-	-	34	-
	**HDDA ^f^**	-	-	-	34
**Photoinitiator**	**IC-184 ^g^**	5	5	5	5
**Additive**	**E-3035 ^h^**	1	1	1	1

^a^: Aliphatic urethane diacrylate. ^b^: Aliphatic urethane hexacrylate. ^c^: IBOA (isobornyl acrylate). ^d^: IBOMA (isobornyl methacrylate). ^e^: TPGDA (tripropylene glycol diacrylate, Miramer M220). ^f^: HDDA (1,6-hexanediol diacrylate, Miramer M200). ^g^: IC-184 (1-hydroxy cyclohexyl phenyl ketone, Irgacure 184). ^h^: E-3035 (organically modified polyether polysiloxane, EFKA-3035).

**Table 3:** LPH & Reduction thickness by Cross-die cup drawing for UV-cured PUA coating films.

No	Sample	Coating System			LPH (mm)	Reduction Thickness (mm)
		Reactive Diluent Functionality	Thickness	Condition		
1	P-IBOA	1	30 μm	O_2_	44.8	0.53~0.67
2	P-IBOMA	1	30 μm	O_2_	45.2	0.51~0.64
3	P-TPGDA	2	30 μm	O_2_	41.1	0.51~0.68
4	P-HDDA	2	30 μm	O_2_	37.5	0.45~0.65

## Data Availability

Not applicable.

## Supplementary Materials

The following supporting information can be downloaded at: https://www.mdpi.com/article/10.3390/polym15040880/s1, Figure S1: Structures of the reactive diluents used in this study, Figure S2: T-bending test on UV-cured PUA coating films, Figure S3: 0~8 mm Erichsen test on UV-cured PUA coating films, Figure S4: 0~500 mm impact resistance test on UV-cured PUA coating films, Figure S5: (A) LPH test and (B) reduction thickness test on UV-cured PUA coating films, Figure S6: (A) Pencil hardness test and (B) UV-light resistance test on UV-cured PUA coating films in the N_2_ atmosphere, Figure S7: T-bending, Erichsen test and impact resistance images of UV-cured PUA coated films in the N_2_ atmosphere, Figure S8: (A) LPH test and (B) reduction thickness test on UV-cured PUA coating films in the N_2_ atmosphere. Figure S9: Formability test: Compression and tension area images of UV-cured PUA coating films in the N_2_ atmosphere, and Figure S10: ASAME strain signatures of UV-cured PUA coating films (A) P-IBOA and (B) P-IBOMA in the N_2_ atmosphere; Table S1: Viscosities of the UV-curable PUA coatings, Table S2: Gel content (wt%) of the UV-cured PUA coating films, and Table S3: LPH and reduction thickness by Cross-die cup drawing in N2 atmosphere.
