# Supplementary material for: Study on Press Formability and Properties of UV-Curable Polyurethane Acrylate Coatings with Different Reactive Diluents
Source: Polymers (Basel). 2023 Feb 10;15(4):880. doi: 10.3390/polym15040880 (PMC9959498; doi:10.3390/polym15040880)
Supplement: Supplementary file 1 [file polymers-15-00880-s001.zip › polymers-2134012-supplementary.pdf]

Study on Press Formability and Properties of UV-curable Polyurethane Acrylate Coatings with Different Reactive Diluents

Woo-Chan Choi [1, †], Vishal Gavande [a, †], Dong Yun Kim [2], and Won-Ki Lee [2,*]

[1] Central R&D Center, Dongkuk Steel Mill, Nam-gu, Busan, Republic of Korea; woochan.choi@dongkuk.com
[2] Division of Polymer Engineering, Pukyong National University, Busan 48513, Republic of Korea; vgawande77@gmail.com (V.G.); a01084847122@gmail.com (D.-Y.K.)
* Correspondence: wonki@pknu.ac.kr;
† These authors contributed equally to this work.

Supporting Information

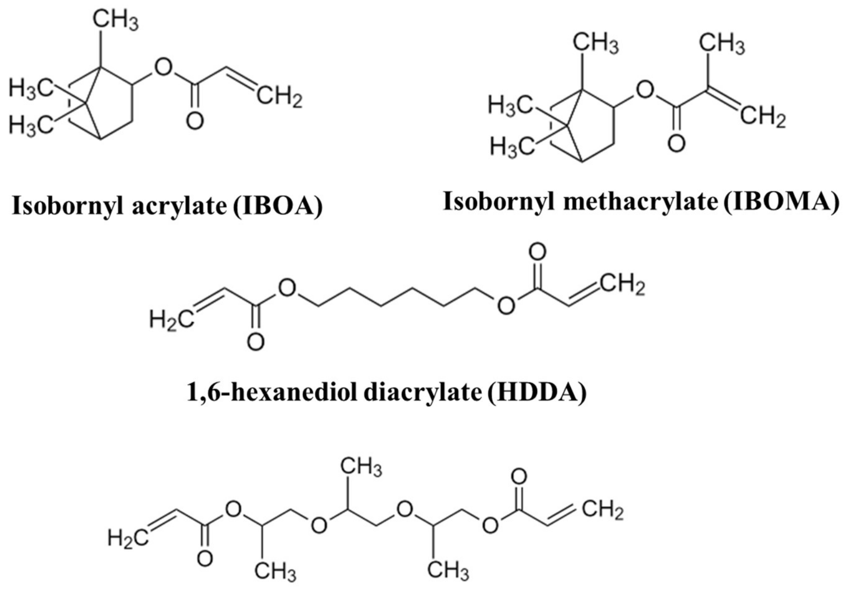

Figure S1. Structures of the reactive diluents used in this study.

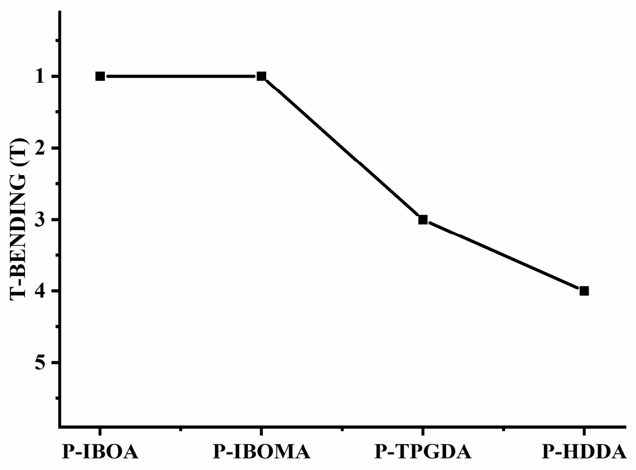

Figure S2. T-bending test on UV-cured PUA coating films.

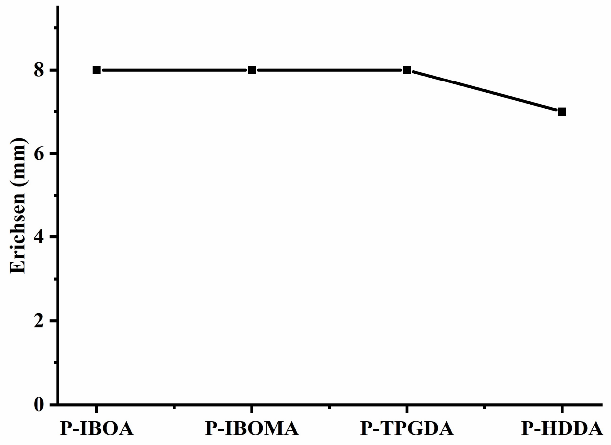

Figure S3. 0~8 mm Erichsen test on UV-cured PUA coating films.

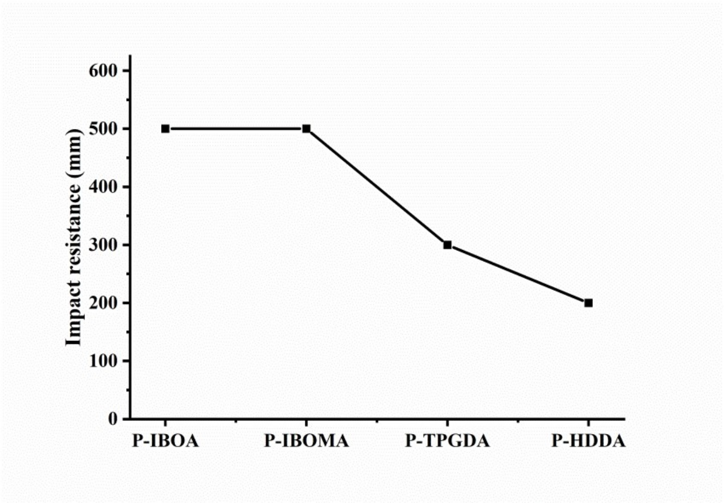

Figure S4. 0~500 mm impact resistance test on UV-cured PUA coating films.

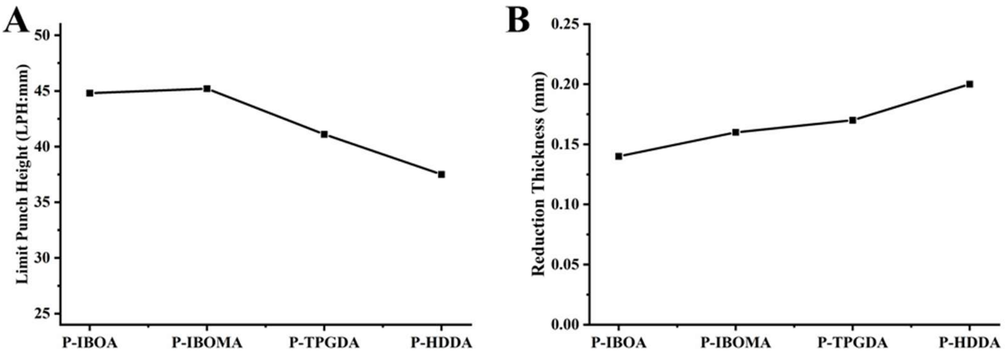

Figure S5. A) LPH test and B) reduction thickness test on UV-cured PUA coating films.

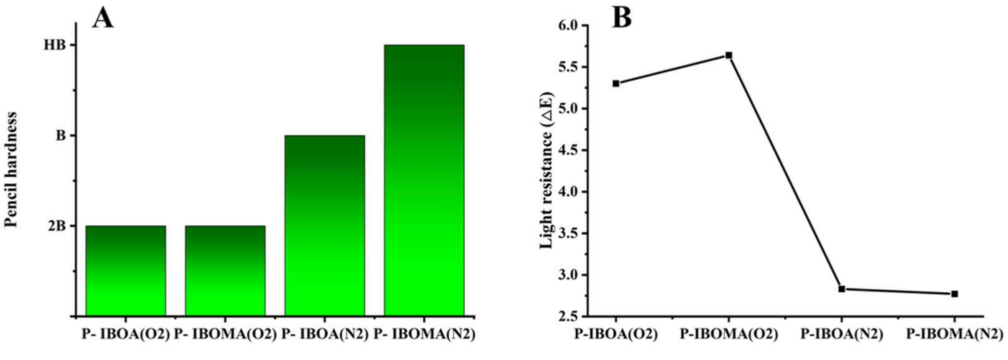

Figure S6. A) Pencil hardness test and B) UV-light resistance test on UV-cured PUA coating films in the N_2 atmosphere.

	**T-bending (1T)**	**8 mm Erichsen**	**500 mm Impact**
**P-IBOA(N_2)**	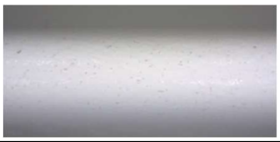	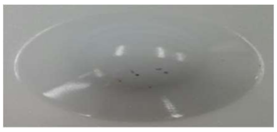	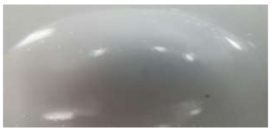
**Result**	GOOD	GOOD	GOOD
	T-bending (1T)	8 mm Erichsen	500 mm Impact
**P-IBOMA(N_2)**	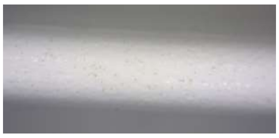	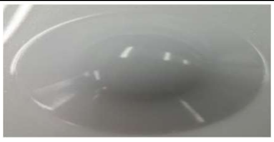	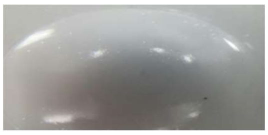
**Result**	GOOD	GOOD	GOOD

Figure S7. T-bending, Erichsen test and impact resistance images of UV-cured PUA coated films in the N_2 atmosphere.

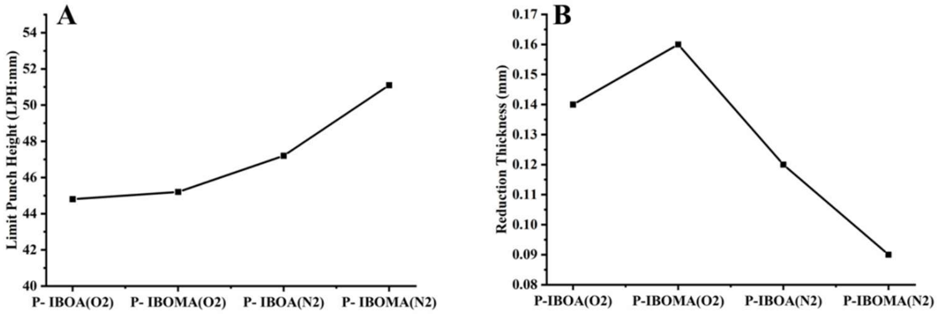

Figure S8. A) LPH test and B) reduction thickness test on UV-cured PUA coating films in the N_2 atmosphere.

	P-IBOA(N_2)	P-IBOMA(N_2)
	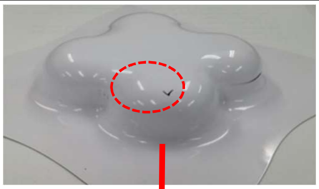	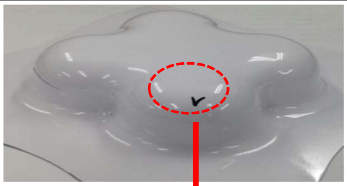
**Compression and tension area**	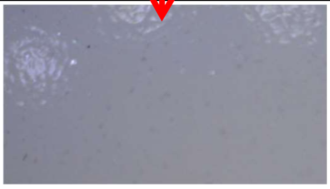	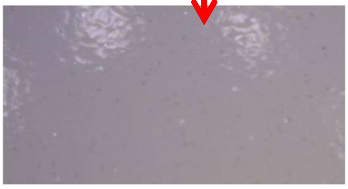
**Result**	GOOD	GOOD

Figure S9. Formability test: Compression and tension area images of UV-cured PUA coating films in the N_2 atmosphere.

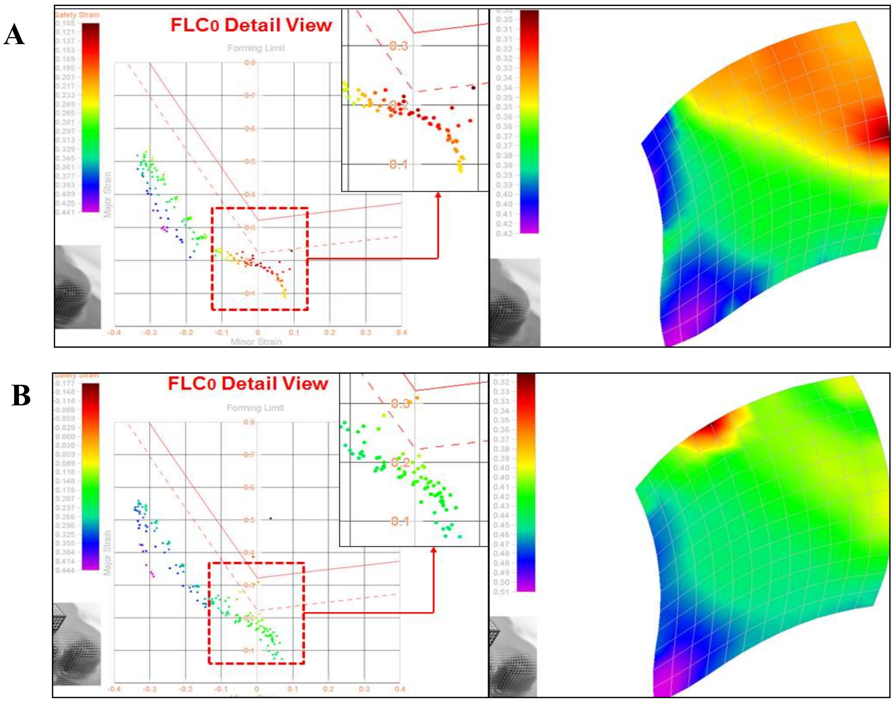

Figure S10. ASAME strain signatures of UV-cured PUA coating films A) P-IBOA and B) P-IBOMA in the N_2 atmosphere.

Table S1. Viscosities of the UV-curable PUA coatings

Oligomer	P-IBOA	P-IBOMA	P-TPGDA	P-HDDA
25500 cps	1080 cps	1120 cps	767 cps	426 cps

Table S2. Gel content (wt%) of the UV-cured PUA coating films

P-IBOA (0_2)	P-IBOMA (0_2)	P-TPGDA (0_2)	P-HDDA (0_2)	P-IBOA (N_2)	P-IBOMA (N_2)
95.8	95.5	96.5	96.9	99.3	99.5

Table S3. LPH and reduction thickness by Cross-die cup drawing in N2 atmosphere

No	Sample	Coating System			LPH (mm)	Reduction thickness (mm)
		Reactive diluent functionality	Thickness	Condition		
**1**	P-IBOA	1	30 μm	N_2	47.2	0.52~0.64
**2**	P-IBOMA	1	30 μm	N_2	51.1	0.52~0.61
